# Grading of prostatic adenocarcinoma: current state and prognostic implications

**DOI:** 10.1186/s13000-016-0478-2

**Published:** 2016-03-09

**Authors:** Jennifer Gordetsky, Jonathan Epstein

**Affiliations:** Department of Pathology, University of Alabama at Birmingham, Birmingham, AL USA; Department of Urology, University of Alabama at Birmingham, Birmingham, AL USA; Department of Pathology, The Johns Hopkins Hospital, The Weinberg Building Room 2242. 401 North Broadway St., Baltimore, MD 21231 USA; Department of Urology, The Johns Hopkins Hospital, Baltimore, MD USA

**Keywords:** Prostate cancer, Grading, Prognosis

## Abstract

**Background:**

Despite significant changes in the clinical and histologic diagnosis of prostate cancer, the Gleason grading system remains one of the most powerful prognostic predictors in prostate cancer. The correct diagnosis and grading of prostate cancer is crucial for a patient’s prognosis and therapeutic options. However, this system has undergone significant revisions and continues to have deficiencies that can potentially impact patient care.

**Main Body:**

We describe the current state of grading prostate cancer, focusing on the current guidelines for the Gleason grading system and recent changes from the 2014 International Society of Urological Pathology Consensus Conference on Gleason Grading of Prostatic Carcinoma. We also explore the limitations of the current Gleason grading system and present a validated alternative to the Gleason score. The new grading system initially described in 2013 in a study from Johns Hopkins Hospital and then validated in a multi-institutional study, includes five distinct Grade Groups based on the modified Gleason score groups. Grade Group 1 = Gleason score ≤6, Grade Group 2 = Gleason score 3 + 4 = 7, Grade Group 3 = Gleason score 4 + 3 = 7, Grade Group 4 = Gleason score 8, Grade Group 5 = Gleason scores 9 and 10.

**Conclusion:**

As this new grading system is simpler and more accurately reflects prostate cancer biology, it is recommended by the World Health Organization (WHO) to be used in conjunction with Gleason grading.

## Background

**Fig. 1:**
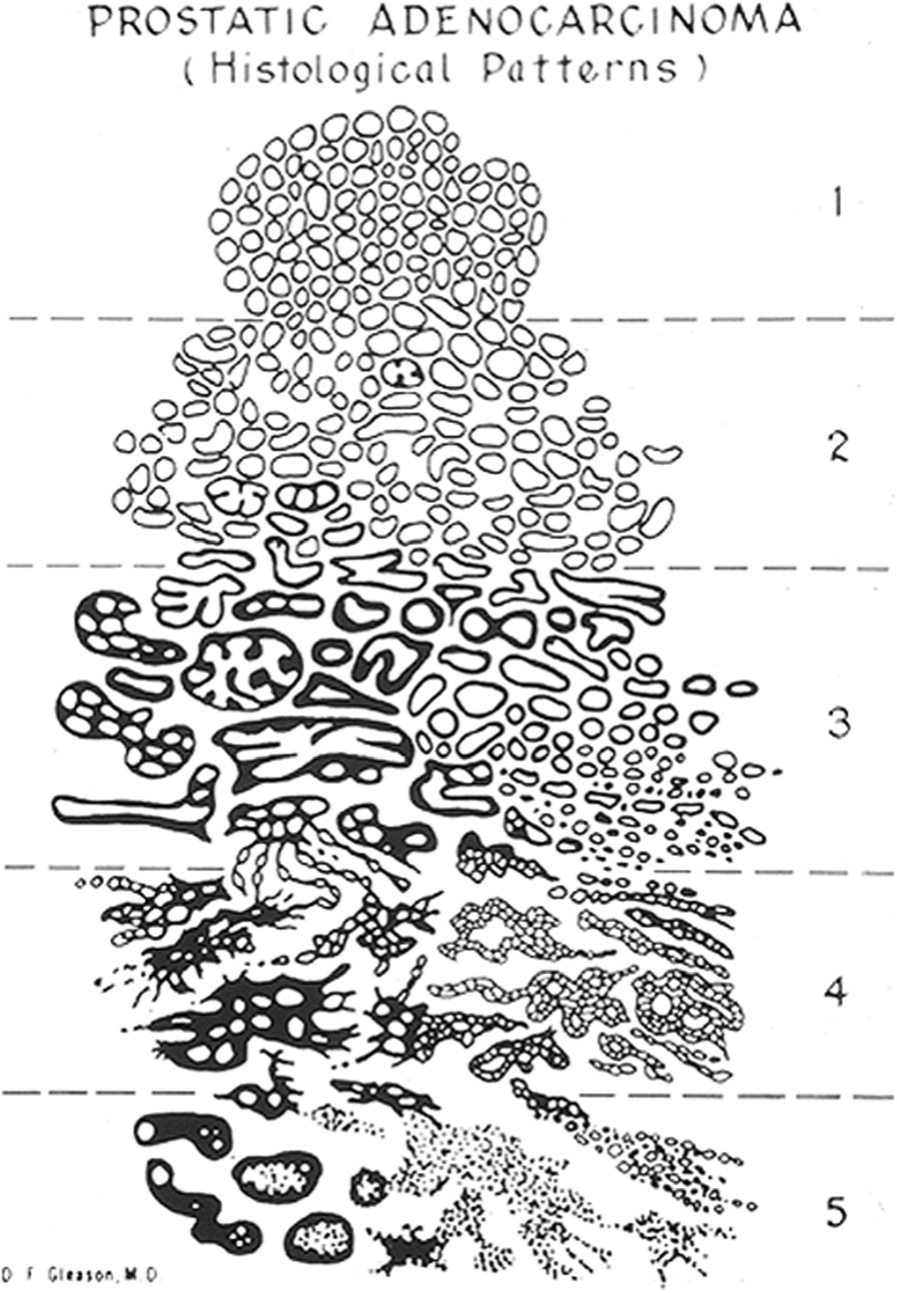
The original Gleason Grading system diagram

The Gleason grading system is based on a study from 1959 through 1964 by the Veteran’s Affairs Cooperative Research Group (VACURG), which enrolled 270 men with prostate cancer [1]. Dr. Donald Gleason, the Chief of Pathology at the Veteran’s Hospital in Minnesota, created a grading system for prostate cancer based on its different histologic patterns. As most of the tumors typically had two histologic patterns, a score was created that added the two most common grade patterns in a tumor, with scores ranging from 2 to 10. The study demonstrated a progressive increase in cancer specific mortality with an increase in score [1]. For ease of grading, the five prognostic patterns were demonstrated by a simple diagram drawn by Dr. Gleason (Fig. 1).

Despite significant changes in the clinical and histologic diagnosis of prostate cancer, the Gleason grading system remains one of the most powerful prognostic predictors in prostate cancer. However, this system has undergone significant revisions and continues to have deficiencies that can potentially impact patient care. Herein we describe the current state of grading prostate cancer, focusing on the current guidelines for the Gleason grading system and recent changes from the 2014 International Society of Urological Pathology (ISUP) Consensus Conference on Gleason Grading of Prostatic Carcinoma [2, 3]. We also explore the limitations of the current Gleason grading system and present a validated alternative to the Gleason score [4, 5].

## Main text

### Gleason grading

Gleason grading of prostatic adenocarcinoma can typically be performed using the 4x objective, although there may be certain instances (ie. back-to-back glands vs. fused glands) that require higher magnification at 10x objective. Gleason scores should be reported as a mathematical equation, for example, Gleason score 4 + 3 = 7, so as to avoid ambiguity.

The current application of the Gleason grading system is significantly different from the original version. Gleason patterns 1 and 2 (Gleason scores 2–5) should no longer be assigned on needle core biopsy. This is because of poor reproducibility and poor correlation with radical prostatectomy grade [6, 7]. In addition, a diagnosis of Gleason score of 2–5 is misleading for both clinicians and patients as nearly all cases show higher grade at resection [6, 7]. The original study by Gleason did not benefit from the use of immunohistochemistry and it is likely that Gleason’s original 1 + 1 = 2 adenocarcinomas were in fact adenosis. In addition, with the current changes in Gleason grading, nearly all the previously considered Gleason pattern 2 adenocarcinomas are now classified as Gleason grade 3. Over the last decade there has been a dramatic decrease in the current incidence of pathologists diagnosing Gleason score 2–4 compared to 22.3 % of the biopsies in 1994 [8, 9].

**Fig. 2:**
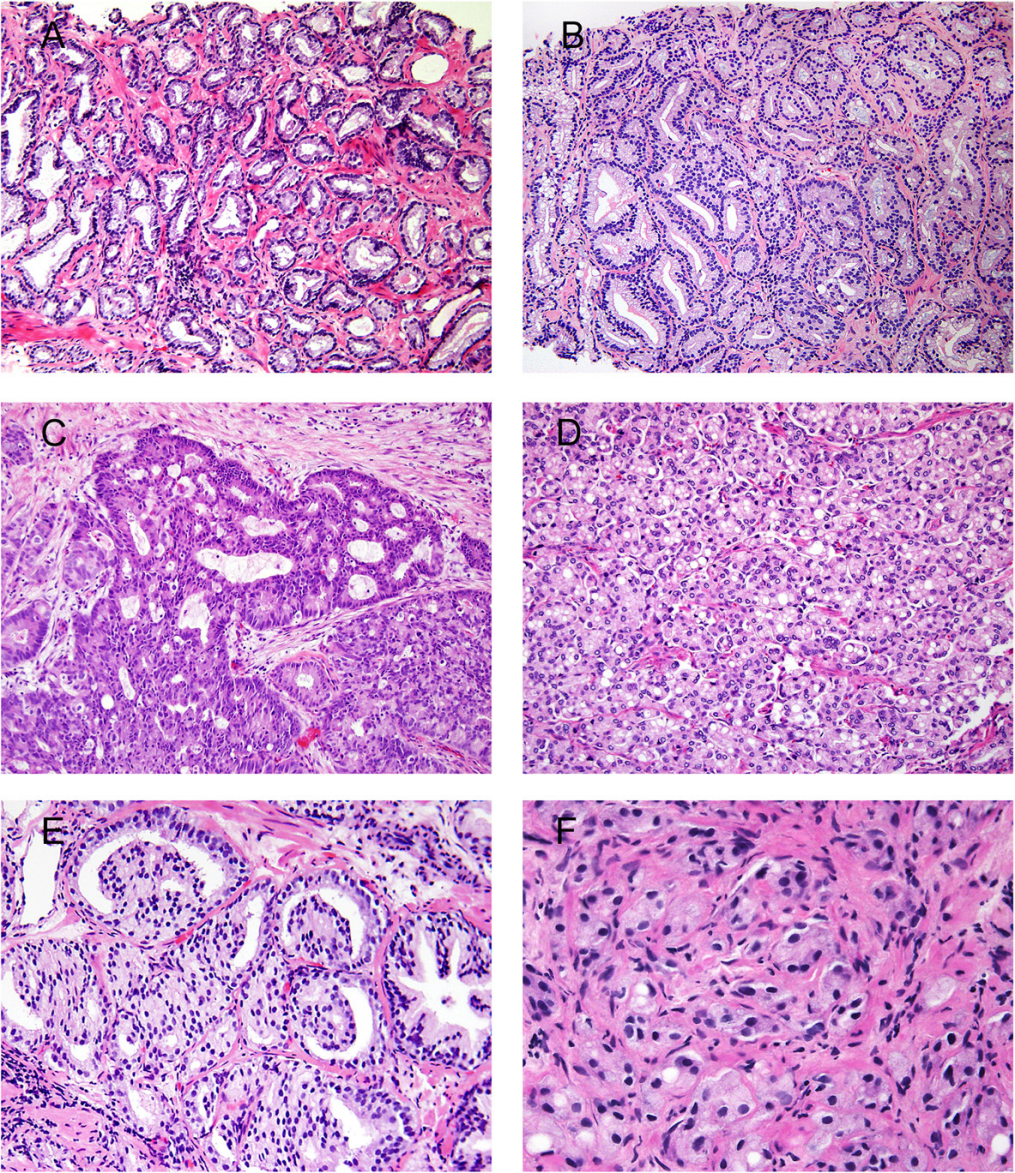
**a** Gleason score 3 + 3 = 6 (Grade Group 1). **b** Gleason score 3 + 4 = 7 (Grade Group 2) with minor component of cribriform glands. **c** Gleason score 4 + 4 = 8 (Grade Group 4) with irregular cribriform glands. **d** Gleason score 4 + 4 = 8 (Grade Group 4) with fused glands with cytoplasmic vacuoles. **e** Gleason score 4 + 4 = 8 (Grade Group 4) with glomeruloid glands. **f** Gleason score 4 + 4 = 8 (Grade Group 4) with poorly-formed glands

Gleason pattern 3 consists of well-formed, individual glands of various sizes including branching glands (Fig. 2a). The glands should form discrete units, such that one could draw a circle around each individual gland. Small glands are acceptable for Gleason grade 3 as long as they are well formed and not fused. Gleason pattern 3 should typically be diagnosed at low magnification (4x objective). This is to prevent over-grading a tumor based on a few poorly formed glands at high power, which could represent tangential sectioning of small well-formed glands. Perineural invasion and mucinous fibroplasia (collagenous micronodules) can also cause glands that appear more complex, and one should be cautious in assigning Gleason pattern 4 in these areas unless overtly cribriform. Biopsies with only a small focus of Gleason pattern 3 can still be assigned a primary and secondary pattern resulting in a Gleason score 3 + 3 = 6, since these biopsies are likely to show Gleason score 3 + 3 = 6 on radical prostatectomy [8].

Gleason pattern 4 includes poorly-formed, fused, and cribriform glands (Fig. 2b-f). Glomeruloid morphology is characterized by dilated glands containing intraluminal cribriform structures with a single point of attachment, resembling a renal glomerulus [10] (Fig. 2e). There was no consensus as to how to grade this particular histologic variant in the 2005 ISUP grading conference. In 2009, Lotan et al. demonstrated that 84 % of cases with glomeruloid glands were associated with Gleason pattern 4 or higher cancer [11]. This same study documented that there were often transitions between small and large glomerulations and cribriform glands. At the 2014 recent ISUP grading conference, it was determined that glomeruloid morphology should be considered Gleason pattern 4 [3]. Cribriform prostate cancer has a spectrum of differentiation. Cribriform glands are classically thought of as having well-formed, punched-out, lumens (Fig. 2c). However, less differentiated examples can have lumina that are not as open but are still considered pattern 4. The original Gleason grading system included round and regular cribriform glands as Gleason pattern 3, whereas in pattern 4 they were more irregular with ragged edges (Fig. 1). Studies have shown that cases with cribriform glands previous considered pattern 3 would uniformly be considered grade 4 by today’s standards [12, 13]. In addition, studies have shown that cribriform pattern in radical prostatectomy specimens are associated with biochemical recurrence, extraprostatic extension, positive surgical margins, distant metastases, and cancer-specific mortality [14–18]. For these reasons, all cribriform glands should be assigned Gleason pattern 4 [3]. The consequence of cribriform glands and poorly-formed glands being considered as Gleason pattern 4 when they were previously grades as Gleason pattern 3 is that there is an increase in Gleason score 7 tumors as well as a better prognosis for current Gleason score 6 tumors compared to their historic counterparts. These modifications have led to a better correlation between the Gleason grade found on biopsy and that found on radical prostatectomy. They have also shown improved prediction of prostatectomy stage, margins, tumor volume, and biochemical recurrence [19–23].

**Fig. 3:**
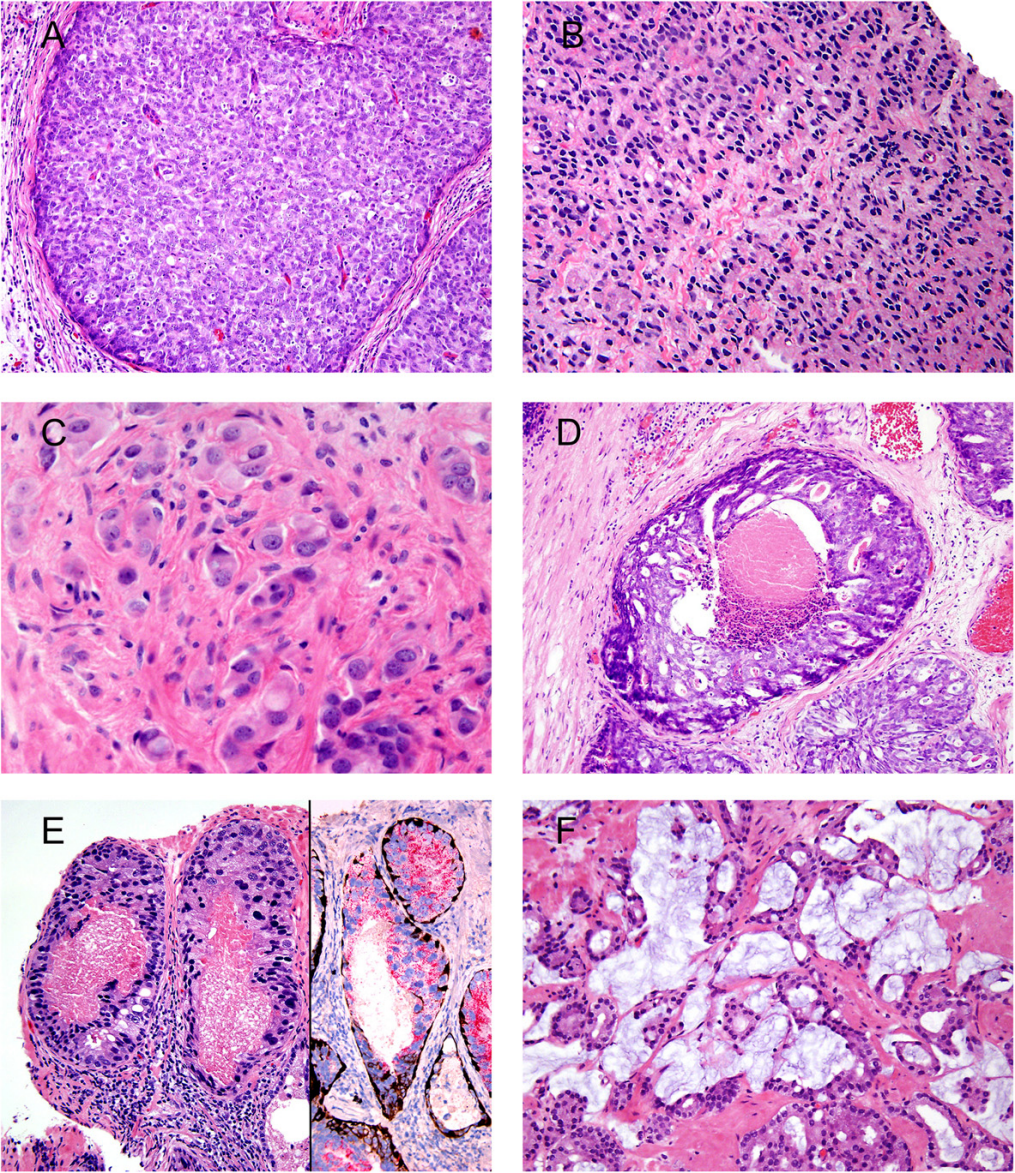
**a** Gleason score 5 + 5 = 10 (Grade Group 5) with solid sheets of cells. **b** Gleason score 5 + 5 = 10 (Grade Group 5) with cords of cells. **c** Gleason score 5 + 5 = 10 (Grade Group 5) with individual cells. **d** Gleason score 5 + 4 = 9 (Grade Group 5) with cribriform glands, some with necrosis. **e** Intraductal carcinoma with necrosis (left), surrounded by basal cells highlighted by p63 and high molecular weight cytokeratin (right) and positive for racemase. **f** Mucinous adenocarcinoma Gleason score 3 + 4 = 7 (Grade Group 2) with individual well-formed glands and minor component of cribriform glands floating in extracellular mucin

Gleason pattern 5 consists of sheets of tumor, individual cells, and cords of cells (Fig. 3a-d). Solid nests of cells with vague microacinar or only occasional gland space formation are also considered Gleason pattern 5. A more uncommon pattern 5 morphology is comedonecrosis within solid nests or cribriform glands. It is important to distinguish intraluminal eosinophilic secretions from true necrosis. Intraductal carcinoma (discussed under histologic variants) can mimic Gleason pattern 5 [24, 25]. Gleason pattern 5 is frequently undergraded by pathologists [26].

### Histologic variants

Intraductal carcinoma (IDC) (Fig. 3e) is the spread of adenocarcinoma within prostatic ducts and in most cases is considered to be extension of high grade invasive tumor into ducts [12, 27]. IDC on needle core biopsy has been shown to be associated with high-grade tumor and high stage disease on radical prostatectomy, metastatic disease, and decreased cancer-free survival [24, 25, 28, 29]. In addition, IDC is an independent prognosticator of early biochemical recurrence and metastases in patients treated with radiation therapy [30]. However, IDC is occasionally a precursor lesion rather than extension of invasive carcinoma into ducts and may be present at radical prostatectomy either without invasive carcinoma or with Gleason score 3 + 3 = 6 cancer [29, 31]. In these cases, if IDC had been assigned a high grade on biopsy it would have been misleading, as pure IDC at radical prostatectomy is thought to have no risk of disease recurrence [29, 25]. In biopsies where IDC is associated with obvious high-grade invasive tumor, immunohistochemistry is not needed for the diagnosis of IDC. Immunohistochemistry should be used in cases where the results of the studies would change the case’s overall grade. At the recent 2014 ISUP grading conference it was recommended that IDC not be graded [3]. However, a comment should be added to the pathology report that this finding is usually associated with high-grade invasive prostate cancer.

Mucinous adenocarcinoma (Fig. 3f) is a variant of prostatic adenocarcinoma where the infiltrative glands have abundant extracellular mucin. There was no consensus as to how to grade this particular histologic variant in the 2005 ISUP grading meeting as the clinical behavior of this entity was unclear. It was previously thought that this tumor behaved as Gleason grade 4 and some proposed to consider all mucinous tumors as Gleason grade 4 [2]. Two studies have since been published that showed mucinous adenocarcinoma of the prostate treated by radical prostatectomy is not more aggressive than non-mucinous prostate cancer [32, 33]. Thus the consensus in the 2014 ISUP grading meeting was to grade this variant based on its underlying growth pattern [3].

### Grading of multiple cores with cancer

In the United States it is standard of care for urologists to perform a 10–12 core biopsy. In some cases, multiple cores may be positive for cancer, with different cores having a different Gleason grade. As long as the cores are submitted in separate containers or designated by location (for example by ink) the pathologist should report the grades of each core separately. In cases where different cores are present within the same specimen container without a designation as to location, there is no consensus whether the individual cores are still given a grade or whether there is only 1 grade given to the part, averaging the grades on different cores. Several studies have demonstrated that in cases with different cores having different grades, the highest Gleason score on a given core correlates better with stage and Gleason score at radical prostatectomy than the average or most frequent grade amongst the cores [34–37]. If multiple fragmented cores are in a specimen container, only an overall Gleason score for that part can be reported. In other countries, there is a tendency to also derive an overall or global grade at the end of the case. An argument against giving an overall (global) grade is that the pathologist cannot know whether the cancer on different cores represents the same or multifocal tumor. The urologist, who can factor in the findings on imaging studies, is better equipped to determine what the overall grade might be in the setting of multifocal tumor. However, in general, the highest grade per core is used to predict prognosis and determine therapy.

### Tertiary grade on needle core biopsy

In contrast to the original Gleason grading system, it is now recommended that on a needle core biopsy both the most common and highest grade are added together for the Gleason score [2]. For example, if there is 60 % Gleason pattern 3, 35 % Gleason pattern 4, and 5 % Gleason pattern 5, the Gleason score would be 3 + 5 = 8. Needle core biopsy is an imperfect, non-targeted, random sampling of the prostate gland. Thus any amount of high-grade tumor sampled on needle biopsy most likely indicates a more significant amount of high-grade tumor within the prostate. In all specimens, in the setting of high-grade cancer, one should not report a lower grade if it occupies less than 5 % of the total tumor. For example, if there is 98 % Gleason pattern 4 and 2 % Gleason pattern 3, the Gleason score would be reported as 4 + 4 = 8 [2].

### Grading on radical prostatectomy

It was recommended at the 2005 ISUP grading conference that each dominant tumor nodule should be given a Gleason Score [2]. This issue becomes important when separate nodules have significantly different Gleason grades. For example, a case might have a nodule of Gleason score 4 + 4 = 8 within the peripheral zone and a larger nodule of Gleason score 3 + 3 = 6 within the transition zone. The patient’s clinical prognosis is going to be dictated by the higher grade tumor, whereas giving an overall Gleason score of 3 + 4 = 7 will undergrade the tumor and be misleading. In order to grade separate dominant tumor nodules, radical prostatectomy specimens should be processed in an organized fashion. This does not necessarily require embedding the entire specimen. However, it does require that the prostate be submitted in a fashion that maintains orientation in order to distinguish between different tumor nodules [38–40]. In most prostates there is one or two dominant nodules. The dominant nodule is typically the largest tumor and is associated with the highest stage and highest grade. Small foci of Gleason score 6 cancer that often co-exist with dominant tumor nodules do not need to be reported. Tertiary grades are provided only in radical prostatectomy specimens, when in a nodule there is a third component of a Gleason pattern higher than the primary and secondary patterns, and where the tertiary component is <5 % of the whole tumor. When the 3rd most common component is the highest grade and occupies >5 % of the tumor, it is typically recorded as the secondary pattern. So 50 % pattern 4, 30 % pattern 3, and 20 % pattern 5, would be reported as 4 + 5 = 9. Tertiary Gleason patterns are in general associated with higher pathological stage and biochemical recurrence as compared to the same Gleason score cancers without tertiary patterns [41–43].

### Clinical risk stratification

Gleason score continues to be the single most powerful predictor of prostate cancer prognosis and directs clinical management. Various scores have been grouped together based on the assumption that they have a similar prognosis. Urologists use Gleason score along with other clinical variables to create risk stratification for patient management. There is considerable diversity in the literature regarding Gleason score grouping including: 2–4, 5–7, 8–10; 2–6, 7, 8–10; and 2–6, 7–10 [44–46]. The most common risk stratification for prostate cancer is the National Comprehensive Cancer Network (NCCN) and D’Amico classification system [47]. It stratifies prostate cancer based on PSA, clinical stage, and biopsy score into low risk (2–6), intermediate risk (7), and high risk (8–10), where both 3 + 4 = 7 and 4 + 3 = 7 is considered the same within the intermediate risk group. Similarly, Gleason score 8 is not distinguished from Gleason score 9–10 in the high risk group. Several studies have shown an adverse prognosis associated with Gleason grade 5, especially as it applies to radiation therapy [48–52].

### Limitations of the gleason grading system

Although current revisions have improved the Gleason grading system, it continues to have limitations. Recent modifications have made the Gleason grading system much more complex than its original version. This complexity can be confusing for patients and clinicians. Gleason score 6 is now recommended as the lowest grade to be assigned on prostate biopsy. This is counterintuitive in that the Gleason scale ranges from 2 to 10. Patients may assume that a diagnosis of Gleason score 6 on biopsy means their tumor is in the mid-range of aggressiveness rather than having the best prognosis. In addition, many former Gleason score 6 tumors are now reclassified as Gleason score 7 in the modified system. Modern Gleason score 6 tumors have a much better prognosis than reported in the older literature. Studies have shown that virtually no pure Gleason score 6 tumors are associated with disease recurrence after radical prostatectomy and pure Gleason 6 cancer at radical prostatectomy lacks the potential for lymph node metastases [4, 53]. Another problem in the modern Gleason grading system is the lumping of Gleason score 7, as noted above in the NCCN Risk Classification System. Whereas many clinicians consider Gleason score 7 on biopsy to be intermediate risk, multiple studies have shown that Gleason score 4 + 3 = 7 demonstrates worse pathological stage and biochemical recurrence rates than 3 + 4 = 7 [54–56].

### A new grading system

In 2013, a new grading system was proposed by the group from Johns Hopkins Hospital [4]. The grading system includes five distinct Grade Groups based on the modified Gleason score groups. Grade Group 1 = Gleason score ≤ 6, Grade Group 2 = Gleason score 3 + 4 = 7, Grade Group 3 = Gleason score 4 + 3 = 7, Grade Group 4 = Gleason score 4 + 4 = 8, Grade Group 5 = Gleason scores 9 and 10. A multi-institutional study including Johns Hopkins Hospital, Memorial Sloan-Kettering Cancer Center (MSKCC), University of Pittsburgh, Cleveland Clinic, and the Karolinska Institute validated the new grading system on 20,845 radical prostatectomy cases with a mean follow-up period, without progression, of 3 years. [5]. The 5-year biochemical risk-free survivals for the 5 Grade Groups based on radical prostatectomy grade were 96, 88, 63, 48, and 26 %, respectively. These Grade Groups were shown to be more accurate in predicting progression than the Gleason risk stratification groups (≤6, 7, 8–10). The Grade Groups showed similar prognostic curves on biopsy in men treated with radiation +/− hormonal therapy as well as radical prostatectomy. Using this new system, patients could be reassured that they have a Grade Group 1 tumor on biopsy that is the lowest grade tumor possible, which in most cases can be followed with active surveillance. Follow-up is still needed as there is unsampled higher grade cancer in approximately 20 % of cases [57]. As this new grading system is simpler and more accurately reflects prostate cancer biology, we recommend using it in conjunction with Gleason grading. For example: Gleason score 3 + 3 = 6 (Grade Group 1). This new grading system has been accepted by the World Health Organization (WHO) for the 2016 edition of Pathology and Genetics: Tumours of the Urinary System and Male Genital Organs [3].

## Conclusions

Gleason score continues to be the single most powerful predictor of prostate cancer prognosis and plays a significant role in clinical management. The correct diagnosis and grading of prostate cancer is crucial for a patient’s prognosis and therapeutic options. The 2005 and 2014 ISUP grading consensus conferences have improved the overall Gleason grading system. However, this system continues to have limitations which a new prostate cancer grading system improves upon.

### Ethics approval and consent to participate

Not applicable.

### Consent for publication

Not applicable.

### Availability of data and materials

Not applicable.

## Abbreviations

IDC: Intraductal carcinoma
ISUP: International Society of Urological Pathology
NCCN: National Comprehensive Cancer Network
VACURG: Veteran’s Affairs Cooperative Research Group

## References

[1] Gleason DF, Mellinger GT (1974). Prediction of prognosis for prostatic adenocarcinoma by combined histological grading and clinical staging. J Urol.

[2] Epstein JI, Allsbrook WC, Amin MB, Egevad LL (2005). ISUP Grading Committee. The 2005 International Society of Urological Pathology(ISUP) Consensus Conference on Gleason Grading of Prostatic Carcinoma. Am J Surg Pathol.

[3] Epstein JI, Egevad L, Amin MB, Delahunt B, Srigley JR, Humphrey PA (2016). The 2014 International Society of Urological Pathology (ISUP) Consensus Conference on Gleason Grading of Prostatic Carcinoma: definition of grading patterns and proposal for a new grading system. Am J Surg Pathol.

[4] Pierorazio PM, Walsh PC, Partin AW, Epstein JI (2013). Prognostic Gleason grade grouping: data based on the modified Gleason scoring sys- tem. BJU Int.

[5] Epstein JI, Zelefsky MJ, Sjoberg DD, Nelson JB, Egevad L, Magi-Galluzzi C, Vickers AJ, Parwani AV, Reuter VE, Fine SW, Eastham JA, Wiklund P, Han M, Reddy CA, Ciezki JP, Nyberg T, Klein EA. A Contemporary Prostate Cancer Grading System: A Validated Alternative to the Gleason Score. Eur Urol. 2016;69(3):428-35. 10.1016/j.eururo.2015.06.046 PMC5002992 26166626

[6] Epstein JI (2000). Gleason score 2–4 adenocarcinoma of the prostate on needle biopsy: a diagnosis that should not be made. Am J Surg Pathol.

[7] Cury J, Coelho RF, Srougi M (2008). Well-differentiated prostate cancer in core biopsy specimens may be associated with extraprostatic disease. Sao Paulo Med J.

[8] Steinberg DM, Sauvageot J, Piantadosi S, Epstein JI (1997). Correlation of prostate needle biopsy and radical prostatectomy Gleason grade in academic and community settings. Am J Surg Pathol.

[9] Fine SW, Epstein JI (2008). A contemporary study correlating prostate needle biopsy and radical prostatectomy Gleason score. J Urol.

[10] Baisden BL, Kahane H, Epstein JI (1999). Perineural invasion, mucinous fibroplasia, and glomerulations: diagnostic features of limited cancer on prostate needle biopsy. Am J Surg Pathol.

[11] Lotan TL, Epstein JI (2009). Gleason grading of prostatic adenocarcinoma with glomeruloid features on needle biopsy. Hum Pathol.

[12] McNeal JE, Yemoto CE (1996). Spread of adenocarcinoma within prostatic ducts and acini. morphologic and clinical correlations. Am J Surg Pathol.

[13] Ross HM, Kryvenko ON, Cowan JE, Simko JP, Wheeler TM, Epstein JI (2012). Do adenocarcinomas of the prostate with Gleason score (GS) <6 have the potential to metastasize to lymph nodes?. Am J Surg Pathol.

[14] Iczkowski KA, Torkko KC, Kotnis GR (2011). Digital quantification of five high-grade prostate cancer patterns, including the cribriform pattern, and their association with adverse outcome. Am J Clin Pathol.

[15] Kir G, Sarbay BC, Gumus E, Topal CS (2014). The association of the cribriform pattern with outcome for prostatic adenocarcinomas. Pathol Res Pract.

[16] Sarbay BC, Kir G, Topal CS, Gumus E (2014). Significance of the cribriform pattern in prostatic adenocarcinomas. Pathol Res Pract.

[17] Trudel D, Downes MR, Sykes J, Kron KJ, Trachtenberg J, van der Kwast TH (2014). Prognostic impact of intraductal carcinoma and large cribriform carcinoma architecture after prostatectomy in a contemporary cohort. Eur J Cancer.

[18] Kweldam CF, Wildhagen MF, Steyerberg EW, Bangma CH, van der Kwast TH, van Leenders GJ (2015). Cribriform growth is highly predictive for postoperative metastasis and disease-specific death in gleason score 7 prostate cancer. Mod Pathol.

[19] Billis A, Guimaraes MS, Freitas LL (2008). The impact of the 2005 international society of urological pathology consensus conference on standard Gleason grading of prostatic carcinoma in needle biopsies. J Urol.

[20] Helpap B, Egevad L (2006). The significance of modified Gleason grading of prostatic carcinoma in biopsy and radical prostatectomy specimens. Virchows Arch.

[21] Ozok HU, Sagnak L, Tuygun C (2010). Will the modification of the Gleason grading system affect the urology practice?. Int J Surg Pathol.

[22] Tsivian M, Sun L, Mouraviev V (2009). Changes in Gleason score grading and their effect in predicting outcome after radical prostatectomy. Urology.

[23] Uemura H, Hoshino K, Sasaki T (2009). Usefulness of the 2005 International Society of Urologic Pathology Gleason grading system in prostate biopsy and radical prostatectomy specimens. BJU Int.

[24] Guo CC, Epstein JI (2006). Intraductal carcinoma of the prostate on needle biopsy: Histologic features and clinical significance. Mod Pathol.

[25] Robinson BD, Epstein JI (2010). Intraductal carcinoma of the prostate without invasive carcinoma on needle biopsy: emphasis on radical prostatectomy findings. J Urol.

[26] Fajardo DA, Miyamoto H, Miller JS, Lee TK, Epstein JI (2011). Identification of Gleason pattern 5 on prostatic needle core biopsy: frequency of underdiagnosis and relation to morphology. Am J Surg Pathol.

[27] Kovi J, Jackson MA, Heshmat MY (1985). Ductal spread in prostatic carcinoma. Cancer.

[28] Zhao T, Liao B, Yao J (2015). Is there any prognostic impact of intraductal carcinoma of prostate in initial diagnosed aggressively metastatic prostate cancer?. Prostate.

[29] Watts K, Li J, Magi-Galluzzi C, Zhou M (2013). Incidence and clinicopathological characteristics of intraductal carcinoma detected in prostate biopsies: A prospective cohort study. Histopathology.

[30] Van der Kwast T, Al Daoud N, Collette L (2012). Biopsy diagnosis of intraductal carcinoma is prognostic in intermediate and high risk prostate cancer patients treated by radiotherapy. Eur J Cancer.

[31] Khani F, Epstein JI (2015). Prostate Biopsy Specimens with Gleason 3 + 3 = 6 and Intraductal Carcinoma: Radical Prostatectomy Findings and Clinical Outcomes. Am J Surg Pathol.

[32] Lane BR, Magi-Galluzzi C, Reuther AM, Levin HS, Zhou M, Klein EA (2006). Mucinous adenocarcinoma of the prostate does not confer poor prognosis. Urology.

[33] Osunkoya AO, Nielsen ME, Epstein JI (2008). Prognosis of mucinous adenocarcinoma of the prostate treated by radical prostatectomy: A study of 47 cases. Am J Surg Pathol.

[34] Kunz GM, Epstein JI (2003). Should each core with prostate cancer be assigned a separate gleason score?. Hum Pathol.

[35] Park HK, Choe G, Byun SS, Lee HW, Lee SE, Lee E (2006). Evaluation of concordance of Gleason score between prostatectomy and biopsies that show more than two different Gleason scores in positive cores. Urology.

[36] Poulos CK, Daggy JK, Cheng L (2005). Preoperative prediction of Gleason grade in radical prostatectomy specimens: the influence of different Gleason grades from multiple positive biopsy sites. Mod Pathol.

[37] Kunju LP, Daignault S, Wei JT, Shah RB (2009). Multiple prostate cancer cores with different Gleason grades submitted in the same specimen container without specific site designation: should each core be assigned an individual Gleason score?. Hum Pathol.

[38] Cohen MB, Soloway MS, Murphy WM (1994). Sampling of radical prostatectomy specimens. How much is adequate?. Am J Clin Pathol.

[39] Hall GS, Kramer CE, Epstein JI (1992). Evaluation of radical prostatectomy specimens. A comparative analysis of sampling methods. Am J Surg Pathol.

[40] Sehdev AE, Pan CC, Epstein JI (2001). Comparative analysis of sampling methods for grossing radical prostatectomy specimens performed for nonpalpable (stage T1c) prostatic adenocarcinoma. Hum Pathol.

[41] Turker P, Bas E, Bozkurt S, Günlüsoy B, Sezgin A, Postacı H (2013). Presence of high grade tertiary Gleason pattern upgrades the Gleason sum score and is inversely associated with biochemical recurrence-free survival. Urol Oncol.

[42] Servoll E, Saeter T, Vlatkovic L, Nesland J, Waaler G, Beisland HO (2010). Does a tertiary Gleason pattern 4 or 5 influence the risk of biochemical relapse after radical prostatectomy for clinically localized prostate cancer?. Scand J Urol Nephrol.

[43] Whittemore DE, Hick EJ, Carter MR, Moul JW, Miranda-Sousa AJ, Sexton WJ (2008). Significance of tertiary Gleason pattern 5 in Gleason score 7 radical prostatectomy specimens. J Urol.

[44] Resnick MJ, Koyama T, Fan KH (2013). Long-term functional outcomes after treatment for localized prostate cancer. N Engl J Med.

[45] Wilt TJ, Brawer MK, Jones KM (2012). Radical prostatectomy versus observation for localized prostate cancer. N Engl J Med.

[46] Bill-Axelson A, Holmberg L, Garmo H (2014). Radical prostatectomy or watchful waiting in early prostate cancer. N Engl J Med.

[47] D’Amico AV, Whittington R, Malkowicz SB (1998). Biochemical outcome after radical prostatectomy, external beam radiation ther- apy, or interstitial radiation therapy for clinically localized prostate cancer. JAMA.

[48] Sabolch A, Feng FY, Daignault-Newton S (2011). Gleason pattern 5 is the greatest risk factor for clinical failure and death from prostate cancer after dose-escalated radiation therapy and hormonal ablation. Int J Radiat Oncol Biol Phys.

[49] Stenmark MH, Blas K, Halverson S, Sandler HM, Feng FY, Hamstra DA (2011). Continued benefit to androgen deprivation therapy for prostate cancer patients treated with dose-escalated radiation therapy across multiple definitions of high-risk disease. Int J Radiat Oncol Biol Phys.

[50] Stock RG, Cesaretti JA, Stone NN (2006). Disease-specific survival following the brachytherapy management of prostate cancer. Int J Radiat Oncol Biol Phys.

[51] Sylvester JE, Grimm PD, Wong J, Galbreath RW, Merrick G, Blasko JC (2011). Fifteen-year biochemical relapse-free survival, cause-specific survival, and overall survival following I(125) prostate brachytherapy in clinically localized prostate cancer: Seattle experience. Int J Radiat Oncol Biol Phys.

[52] Stone NN, Stone MM, Rosenstein BS, Unger P, Stock RG (2011). Influence of pretreatment and treatment factors on intermediate to long-term outcome after prostate brachytherapy. J Urol.

[53] Miyamoto H, Hernandez DJ, Epstein JI (2009). A pathological reassessment of organ-confined, Gleason score 6 prostatic adenocarcinomas that progress after radical prostatectomy. Hum Pathol.

[54] Burdick MJ, Reddy CA, Ulchaker J (2009). Comparison of biochemical relapse-free survival between primary Gleason score 3 and primary Gleason score 4 for biopsy Gleason score 7 prostate cancer. Int J Radiat Oncol Biol Phys.

[55] Chan TY, Partin AW, Walsh PC, Epstein JI (2000). Prognostic significance of Gleason score 3 + 4 versus Gleason score 4 + 3 tumor at radical prostatectomy. Urology.

[56] Kang DE, Fitzsimons NJ, Presti JC (2007). Risk stratification of men with Gleason score 7 to 10 tumors by primary and secondary Gleason score: results from the SEARCH database. Urology.

[57] Epstein JI, Feng Z, Trock BJ, Pierorazio PM (2012). Upgrading and downgrading of prostate cancer from biopsy to radical prostatectomy: Incidence and predictive factors using the modified Gleason grading system and factoring in tertiary grades. Eur Urol.

